# Supplementary material for: Single Cell Transcriptomic and Chromatin Profiles Suggest Layer Vb Is the Only Layer With Shared Excitatory Cell Types in the Medial and Lateral Entorhinal Cortex
Source: Front Neural Circuits. 2022 Jan 26;15:806154. doi: 10.3389/fncir.2021.806154 (PMC8826650; doi:10.3389/fncir.2021.806154)
Supplement: Supplementary file 14 [file Data_Sheet_1.ZIP › Inh_vs_Exc_Motifs/knownResults.html]

Inh\_vs\_Exc\_Motifs/ - Homer Known Motif Enrichment Results


# Homer Known Motif Enrichment Results (Inh\_vs\_Exc\_Motifs/)

Homer *de novo* Motif Results  
Gene Ontology Enrichment Results  
Known Motif Enrichment Results (txt file)  
Total Target Sequences = 12243, Total Background Sequences = 38572

|  |  |  |  |  |  |  |  |  |  |  |  |
| --- | --- | --- | --- | --- | --- | --- | --- | --- | --- | --- | --- |
| Rank | Motif | Name | P-value | log P-pvalue | q-value (Benjamini) | # Target Sequences with Motif | % of Targets Sequences with Motif | # Background Sequences with Motif | % of Background Sequences with Motif | Motif File | SVG |
| 1 | T G C A C T G A A T G C G T C A A C G T A T G C A C G T A C T G A C T G T G C A | ZBTB18(Zf)/HEK293-ZBTB18.GFP-ChIP-Seq(GSE58341)/Homer | 1e-81 | -1.887e+02 | 0.0000 | 2833.0 | 23.14% | 6322.0 | 16.39% | motif file (matrix) | svg |
| 2 | A T C G A T G C A G T C T A G C G A C T T C G A G C T A G C A T A G C T C T G A | DLX1(Homeobox)/BasalGanglia-Dlx1-ChIP-seq(GSE124936)/Homer | 1e-78 | -1.807e+02 | 0.0000 | 5760.0 | 47.05% | 14919.1 | 38.68% | motif file (matrix) | svg |
| 3 | C G A T T G C A T G C A G A T C C G T A A C T G T G A C G A C T C A T G A C T G | Tcf21(bHLH)/ArterySmoothMuscle-Tcf21-ChIP-Seq(GSE61369)/Homer | 1e-77 | -1.783e+02 | 0.0000 | 5020.0 | 41.01% | 12700.6 | 32.93% | motif file (matrix) | svg |
| 4 | G T C A T G C A G C T A A G T C C G T A A C T G T G A C G C A T T C A G C A G T | Ap4(bHLH)/AML-Tfap4-ChIP-Seq(GSE45738)/Homer | 1e-76 | -1.760e+02 | 0.0000 | 5752.0 | 46.99% | 14937.5 | 38.73% | motif file (matrix) | svg |
| 5 | C T G A T C A G A G T C C G T A A T C G A T G C C G A T A C T G A G T C G A C T A T C G A G T C | MyoD(bHLH)/Myotube-MyoD-ChIP-Seq(GSE21614)/Homer | 1e-72 | -1.662e+02 | 0.0000 | 4018.0 | 32.82% | 9843.6 | 25.52% | motif file (matrix) | svg |
| 6 | T C A G C T A G A T G C G A C T T G C A C G T A A G C T A C G T C T G A T A C G | En1(Homeobox)/SUM149-EN1-ChIP-Seq(GSE120957)/Homer | 1e-62 | -1.446e+02 | 0.0000 | 6906.0 | 56.41% | 18834.5 | 48.83% | motif file (matrix) | svg |
| 7 | A T C G A T C G A T G C G A C T T C G A C G T A G C A T A G C T C T G A T A C G | DLX2(Homeobox)/BasalGanglia-Dlx2-ChIP-seq(GSE124936)/Homer | 1e-59 | -1.368e+02 | 0.0000 | 6188.0 | 50.55% | 16663.6 | 43.20% | motif file (matrix) | svg |
| 8 | C A T G A G T C A G C T C G T A C G A T C G A T G C A T G C A T C G A T C T G A C A T G T G A C | Mef2d(MADS)/Retina-Mef2d-ChIP-Seq(GSE61391)/Homer | 1e-58 | -1.339e+02 | 0.0000 | 1748.0 | 14.28% | 3738.7 | 9.69% | motif file (matrix) | svg |
| 9 | A T G C T A C G A G C T T G C A C G T A C G A T A C G T C T G A | DLX5(Homeobox)/BasalGanglia-Dlx5-ChIP-seq(GSE124936)/Homer | 1e-55 | -1.275e+02 | 0.0000 | 3918.0 | 32.00% | 9889.9 | 25.64% | motif file (matrix) | svg |
| 10 | C A T G A G T C G A C T C G T A C G A T G C A T G A C T G C A T C G A T C T A G C A T G T G A C | Mef2b(MADS)/HEK293-Mef2b.V5-ChIP-Seq(GSE67450)/Homer | 1e-54 | -1.259e+02 | 0.0000 | 4853.0 | 39.64% | 12688.7 | 32.90% | motif file (matrix) | svg |
| 11 | C A T G G T A C G A C T G C T A C G T A C G T A C G T A G C T A G A C T C T G A T C A G G T A C | Mef2c(MADS)/GM12878-Mef2c-ChIP-Seq(GSE32465)/Homer | 1e-52 | -1.214e+02 | 0.0000 | 3172.0 | 25.91% | 7777.4 | 20.16% | motif file (matrix) | svg |
| 12 | G T A C G A C T C G T A C T G A T C G A C G T A G C T A C A G T C T G A T A C G | Mef2a(MADS)/HL1-Mef2a.biotin-ChIP-Seq(GSE21529)/Homer | 1e-49 | -1.147e+02 | 0.0000 | 2828.0 | 23.10% | 6852.3 | 17.77% | motif file (matrix) | svg |
| 13 | C A T G A G C T T A C G G T C A G T A C T A G C A G C T G A C T A T C G T C G A | Esrrb(NR)/mES-Esrrb-ChIP-Seq(GSE11431)/Homer | 1e-48 | -1.114e+02 | 0.0000 | 2206.0 | 18.02% | 5132.4 | 13.31% | motif file (matrix) | svg |
| 14 | C G T A C G T A A G C T G A C T T G C A G T C A A C G T A G C T C T G A T C A G | Lhx3(Homeobox)/Neuron-Lhx3-ChIP-Seq(GSE31456)/Homer | 1e-45 | -1.054e+02 | 0.0000 | 6289.0 | 51.37% | 17331.7 | 44.94% | motif file (matrix) | svg |
| 15 | A C T G C A G T A C G T C G T A C G T A A C G T A C T G C T G A | Nkx6.1(Homeobox)/Islet-Nkx6.1-ChIP-Seq(GSE40975)/Homer | 1e-44 | -1.023e+02 | 0.0000 | 8215.0 | 67.11% | 23517.5 | 60.97% | motif file (matrix) | svg |
| 16 | C A T G G A C T T A C G G T C A G T A C G A T C G A C T A G C T A T C G T C G A T A C G T A G C | ERRg(NR)/Kidney-ESRRG-ChIP-Seq(GSE104905)/Homer | 1e-43 | -9.970e+01 | 0.0000 | 2659.0 | 21.72% | 6503.9 | 16.86% | motif file (matrix) | svg |
| 17 | T C G A A G T C C G T A A T C G A T G C C G A T A C T G A G T C A G C T A C T G | Tcf12(bHLH)/GM12878-Tcf12-ChIP-Seq(GSE32465)/Homer | 1e-38 | -8.853e+01 | 0.0000 | 4717.0 | 38.53% | 12696.4 | 32.92% | motif file (matrix) | svg |
| 18 | T G A C C G T A C T G A A C T G A C T G G A C T G A T C T G C A G T A C T A C G | SF1(NR)/H295R-Nr5a1-ChIP-Seq(GSE44220)/Homer | 1e-37 | -8.577e+01 | 0.0000 | 1671.0 | 13.65% | 3856.6 | 10.00% | motif file (matrix) | svg |
| 19 | A C G T T C G A T C G A A G T C G T C A T A C G A T G C A C G T A C T G A G C T | Myf5(bHLH)/GM-Myf5-ChIP-Seq(GSE24852)/Homer | 1e-36 | -8.514e+01 | 0.0000 | 3800.0 | 31.04% | 9981.3 | 25.88% | motif file (matrix) | svg |
| 20 | T C G A C A G T A C T G A G C T C G T A C G T A A C G T A C G T C T G A T A G C | Dlx3(Homeobox)/Kerainocytes-Dlx3-ChIP-Seq(GSE89884)/Homer | 1e-35 | -8.262e+01 | 0.0000 | 3394.0 | 27.72% | 8808.8 | 22.84% | motif file (matrix) | svg |
| 21 | T C G A T C A G T C G A A C T G C A T G A C G T A G T C C T G A | COUP-TFII(NR)/Artia-Nr2f2-ChIP-Seq(GSE46497)/Homer | 1e-35 | -8.257e+01 | 0.0000 | 4207.0 | 34.37% | 11229.9 | 29.12% | motif file (matrix) | svg |
| 22 | T C A G T C A G A C G T G T A C G C T A T C A G C T G A A C T G A C T G A G C T A G T C C G T A | EAR2(NR)/K562-NR2F6-ChIP-Seq(Encode)/Homer | 1e-34 | -7.883e+01 | 0.0000 | 3381.0 | 27.62% | 8813.6 | 22.85% | motif file (matrix) | svg |
| 23 | G T A C C G T A C G T A T A C G G C A T G T A C C G T A C A T G A G T C C G T A C G T A C G A T G C A T G C A T G A C T | MafF(bZIP)/HepG2-MafF-ChIP-Seq(GSE31477)/Homer | 1e-33 | -7.649e+01 | 0.0000 | 1421.0 | 11.61% | 3245.2 | 8.41% | motif file (matrix) | svg |
| 24 | A T G C T A C G A G T C G A C T T G C A C T G A G A C T A C G T C T G A T C A G | LHX9(Homeobox)/Hct116-LHX9.V5-ChIP-Seq(GSE116822)/Homer | 1e-32 | -7.533e+01 | 0.0000 | 5630.0 | 45.99% | 15671.7 | 40.63% | motif file (matrix) | svg |
| 25 | A C G T G A C T T A G C C G T A C T G A C A T G C T A G G A C T G A T C C G T A | Nr5a2(NR)/Pancreas-LRH1-ChIP-Seq(GSE34295)/Homer | 1e-32 | -7.488e+01 | 0.0000 | 2372.0 | 19.38% | 5920.6 | 15.35% | motif file (matrix) | svg |
| 26 | A C G T G A C T A T G C G C T A C T G A C T A G A C T G G A C T A G T C C G T A | Nr5a2(NR)/mES-Nr5a2-ChIP-Seq(GSE19019)/Homer | 1e-32 | -7.440e+01 | 0.0000 | 1845.0 | 15.07% | 4432.2 | 11.49% | motif file (matrix) | svg |
| 27 | T A C G A C T G A G C T G T A C C G T A T C G A C T G A A C T G C A T G A C G T A G T C C G T A | COUP-TFII(NR)/K562-NR2F1-ChIP-Seq(Encode)/Homer | 1e-32 | -7.386e+01 | 0.0000 | 3700.0 | 30.22% | 9819.7 | 25.46% | motif file (matrix) | svg |
| 28 | G A C T C T A G G A T C C A G T A C T G C T G A A T G C G C A T A T G C C T G A | MafA(bZIP)/Islet-MafA-ChIP-Seq(GSE30298)/Homer | 1e-30 | -6.989e+01 | 0.0000 | 3474.0 | 28.38% | 9196.3 | 23.84% | motif file (matrix) | svg |
| 29 | A G C T G T C A C G T A A C G T A C G T C T G A T C A G A T G C | Lhx2(Homeobox)/HFSC-Lhx2-ChIP-Seq(GSE48068)/Homer | 1e-30 | -6.952e+01 | 0.0000 | 4643.0 | 37.93% | 12720.4 | 32.98% | motif file (matrix) | svg |
| 30 | C G T A G A C T C G A T A T C G G T A C G C A T C A T G C G T A T A C G G C A T G T A C C G T A C A T G A T G C G C T A C T A G G C A T G C A T G C A T G A C T | MafB(bZIP)/BMM-Mafb-ChIP-Seq(GSE75722)/Homer | 1e-28 | -6.586e+01 | 0.0000 | 2088.0 | 17.06% | 5200.1 | 13.48% | motif file (matrix) | svg |
| 31 | C G A T T A C G T G A C G A C T C A T G C G T A T A C G A C G T G T A C C T G A | Bach2(bZIP)/OCILy7-Bach2-ChIP-Seq(GSE44420)/Homer | 1e-21 | -4.970e+01 | 0.0000 | 1145.0 | 9.35% | 2707.9 | 7.02% | motif file (matrix) | svg |
| 32 | G C T A C G T A A G T C A C G T T C G A T A C G A C T G A G C T A G T C T C G A | RORgt(NR)/EL4-RORgt.Flag-ChIP-Seq(GSE56019)/Homer | 1e-21 | -4.960e+01 | 0.0000 | 630.0 | 5.15% | 1328.2 | 3.44% | motif file (matrix) | svg |
| 33 | G C T A C G T A A G T C A C G T T C G A T A C G A C T G A G C T A G T C T C G A | RORgt(NR)/EL4-RORgt.Flag-ChIP-Seq(GSE56019)/Homer | 1e-21 | -4.960e+01 | 0.0000 | 630.0 | 5.15% | 1328.2 | 3.44% | motif file (matrix) | svg |
| 34 | T C G A T C G A A G T C C G T A C T A G T A G C A C G T A C T G | MyoG(bHLH)/C2C12-MyoG-ChIP-Seq(GSE36024)/Homer | 1e-21 | -4.954e+01 | 0.0000 | 4825.0 | 39.41% | 13584.7 | 35.22% | motif file (matrix) | svg |
| 35 | C G T A C A T G A G T C G A C T T G C A C G T A A C G T A C G T C T G A T C A G | Lhx1(Homeobox)/EmbryoCarcinoma-Lhx1-ChIP-Seq(GSE70957)/Homer | 1e-19 | -4.462e+01 | 0.0000 | 4699.0 | 38.38% | 13283.0 | 34.44% | motif file (matrix) | svg |
| 36 | C G T A C G T A C G T A A G T C A G C T C T G A A C T G A C T G A G C T A G T C C G T A C T A G C T A G C T A G T G C A | RORa(NR)/Liver-Rora-ChIP-Seq(GSE101115)/Homer | 1e-18 | -4.212e+01 | 0.0000 | 689.0 | 5.63% | 1532.8 | 3.97% | motif file (matrix) | svg |
| 37 | T A G C C T A G T C G A G A C T A C T G C T G A A G T C T C A G G C A T T G A C C T G A A G C T | Atf7(bZIP)/3T3L1-Atf7-ChIP-Seq(GSE56872)/Homer | 1e-17 | -3.994e+01 | 0.0000 | 1740.0 | 14.21% | 4492.4 | 11.65% | motif file (matrix) | svg |
| 38 | T C A G T A G C G A C T C A T G C T G A A T C G G C A T G T A C C G T A A C T G T A G C T G C A | MafK(bZIP)/C2C12-MafK-ChIP-Seq(GSE36030)/Homer | 1e-17 | -3.950e+01 | 0.0000 | 1137.0 | 9.29% | 2780.2 | 7.21% | motif file (matrix) | svg |
| 39 | C G T A C G T A C G T A A G T C A G C T C T G A A C T G C T A G A G C T A G T C C G T A T C A G | RORg(NR)/Liver-Rorc-ChIP-Seq(GSE101115)/Homer | 1e-16 | -3.758e+01 | 0.0000 | 496.0 | 4.05% | 1056.2 | 2.74% | motif file (matrix) | svg |
| 40 | T C G A G C A T A C T G C T G A A G T C T C A G G A C T G T A C C G T A A G C T A G T C G A T C | c-Jun-CRE(bZIP)/K562-cJun-ChIP-Seq(GSE31477)/Homer | 1e-16 | -3.692e+01 | 0.0000 | 1182.0 | 9.66% | 2933.9 | 7.61% | motif file (matrix) | svg |
| 41 | A T G C T C A G T C G A G C A T A C T G C G T A A G T C T C A G G A C T T G A C C G T A A G C T | Atf2(bZIP)/3T3L1-Atf2-ChIP-Seq(GSE56872)/Homer | 1e-14 | -3.358e+01 | 0.0000 | 1250.0 | 10.21% | 3163.2 | 8.20% | motif file (matrix) | svg |
| 42 | A G T C C G A T C T G A C G T A A C G T C A G T T C A G T G A C | Isl1(Homeobox)/Neuron-Isl1-ChIP-Seq(GSE31456)/Homer | 1e-14 | -3.313e+01 | 0.0000 | 6325.0 | 51.67% | 18573.4 | 48.15% | motif file (matrix) | svg |
| 43 | T C G A A C G T A C T G C T G A A G T C T C A G A G C T G T A C C G T A A G C T G A T C T C G A | JunD(bZIP)/K562-JunD-ChIP-Seq/Homer | 1e-13 | -3.085e+01 | 0.0000 | 427.0 | 3.49% | 920.4 | 2.39% | motif file (matrix) | svg |
| 44 | T A C G T C G A C A G T A C T G G C T A A T G C C G A T G T A C C G T A A C T G T A G C C G T A | NF-E2(bZIP)/K562-NFE2-ChIP-Seq(GSE31477)/Homer | 1e-12 | -2.978e+01 | 0.0000 | 392.0 | 3.20% | 836.6 | 2.17% | motif file (matrix) | svg |
| 45 | C T A G T C G A A C G T A C T G C G T A T A G C C G A T G T A C C G T A A G C T G A T C G T A C | Jun-AP1(bZIP)/K562-cJun-ChIP-Seq(GSE31477)/Homer | 1e-12 | -2.965e+01 | 0.0000 | 1412.0 | 11.53% | 3677.2 | 9.53% | motif file (matrix) | svg |
| 46 | A G T C T G C A T C G A C T G A A C T G C A T G A C G T A T G C G T C A T A C G | Erra(NR)/HepG2-Erra-ChIP-Seq(GSE31477)/Homer | 1e-12 | -2.780e+01 | 0.0000 | 5419.0 | 44.27% | 15857.5 | 41.11% | motif file (matrix) | svg |
| 47 | C T A G T C G A C G A T A C T G C G T A T A C G A G C T T G A C G C T A A C G T G A T C T A G C | Fosl2(bZIP)/3T3L1-Fosl2-ChIP-Seq(GSE56872)/Homer | 1e-12 | -2.771e+01 | 0.0000 | 1792.0 | 14.64% | 4814.2 | 12.48% | motif file (matrix) | svg |
| 48 | C G T A C G T A C G T A G C A T G C A T T A C G G T A C G A C T A C T G C G T A A T C G A C G T G T A C C G T A A G C T | Bach1(bZIP)/K562-Bach1-ChIP-Seq(GSE31477)/Homer | 1e-10 | -2.461e+01 | 0.0000 | 381.0 | 3.11% | 843.0 | 2.19% | motif file (matrix) | svg |
| 49 | G C A T T C A G C T G A A T C G A C T G C G A T G A T C C T G A | THRb(NR)/Liver-NR1A2-ChIP-Seq(GSE52613)/Homer | 1e-10 | -2.375e+01 | 0.0000 | 8568.0 | 69.99% | 25942.4 | 67.26% | motif file (matrix) | svg |
| 50 | T C G A A C G T C A T G G C T A T A G C C G A T G T A C G C T A A C G T A T G C | AP-1(bZIP)/ThioMac-PU.1-ChIP-Seq(GSE21512)/Homer | 1e-10 | -2.370e+01 | 0.0000 | 3381.0 | 27.62% | 9664.5 | 25.06% | motif file (matrix) | svg |
| 51 | C A T G C T A G T C G A A C G T A C T G C G T A T A G C C G A T T G A C C G T A A G C T G A T C | Fra2(bZIP)/Striatum-Fra2-ChIP-Seq(GSE43429)/Homer | 1e-10 | -2.325e+01 | 0.0000 | 2433.0 | 19.87% | 6801.4 | 17.63% | motif file (matrix) | svg |
| 52 | C T A G T C G A G C A T C A T G G C T A T A G C C G A T G T A C C T G A A G C T | JunB(bZIP)/DendriticCells-Junb-ChIP-Seq(GSE36099)/Homer | 1e-9 | -2.202e+01 | 0.0000 | 2666.0 | 21.78% | 7529.5 | 19.52% | motif file (matrix) | svg |
| 53 | T C A G A C G T A G T C T C G A A G T C T C A G G C A T C T A G C T A G A G C T | Usf2(bHLH)/C2C12-Usf2-ChIP-Seq(GSE36030)/Homer | 1e-9 | -2.170e+01 | 0.0000 | 1126.0 | 9.20% | 2959.7 | 7.67% | motif file (matrix) | svg |
| 54 | G T C A G C A T A C T G G T A C G A C T A C T G G C T A A T C G C A G T G T A C C G T A A G C T | Nrf2(bZIP)/Lymphoblast-Nrf2-ChIP-Seq(GSE37589)/Homer | 1e-9 | -2.104e+01 | 0.0000 | 316.0 | 2.58% | 697.0 | 1.81% | motif file (matrix) | svg |
| 55 | A C T G C A T G T C G A A C G T A C T G C G T A A T C G A C G T G T A C C G T A G A C T A G T C | Fos(bZIP)/TSC-Fos-ChIP-Seq(GSE110950)/Homer | 1e-8 | -2.039e+01 | 0.0000 | 2879.0 | 23.52% | 8211.2 | 21.29% | motif file (matrix) | svg |
| 56 | T G C A T C G A T C G A A C G T C A T G C G T A A G T C T C A G A C G T G T A C C G T A A G C T | CREB5(bZIP)/LNCaP-CREB5.V5-ChIP-Seq(GSE137775)/Homer | 1e-8 | -2.038e+01 | 0.0000 | 1539.0 | 12.57% | 4189.7 | 10.86% | motif file (matrix) | svg |
| 57 | T A C G T C G A G A C T A C T G C T G A A G T C T C A G G A C T T G A C C T G A | Atf1(bZIP)/K562-ATF1-ChIP-Seq(GSE31477)/Homer | 1e-8 | -2.009e+01 | 0.0000 | 2319.0 | 18.94% | 6523.1 | 16.91% | motif file (matrix) | svg |
| 58 | A C T G C T A G T C G A C G A T C A T G G C T A A T C G C G A T G T A C G C T A A G C T G T A C | Fra1(bZIP)/BT549-Fra1-ChIP-Seq(GSE46166)/Homer | 1e-7 | -1.833e+01 | 0.0000 | 2717.0 | 22.19% | 7767.3 | 20.14% | motif file (matrix) | svg |
| 59 | C T A G T C G A A C G T A C T G C G T A A T G C A C G T G T A C C G T A A G C T G A T C G T A C | Atf3(bZIP)/GBM-ATF3-ChIP-Seq(GSE33912)/Homer | 1e-7 | -1.829e+01 | 0.0000 | 3082.0 | 25.18% | 8880.3 | 23.02% | motif file (matrix) | svg |
| 60 | C T A G T A C G G A T C G T A C G C T A A G C T A G C T G T C A T C G A T A G C | Nanog(Homeobox)/mES-Nanog-ChIP-Seq(GSE11724)/Homer | 1e-7 | -1.706e+01 | 0.0000 | 9985.0 | 81.56% | 30712.2 | 79.63% | motif file (matrix) | svg |
| 61 | C A G T T G C A A C G T A C T G C G T A A T C G C G A T T G A C C G T A A C G T | BATF(bZIP)/Th17-BATF-ChIP-Seq(GSE39756)/Homer | 1e-7 | -1.684e+01 | 0.0000 | 3038.0 | 24.82% | 8784.0 | 22.77% | motif file (matrix) | svg |
| 62 | T C A G A G C T A T G C C G T A A G C T T C A G C A G T A C T G C T G A A G T C | MITF(bHLH)/MastCells-MITF-ChIP-Seq(GSE48085)/Homer | 1e-7 | -1.628e+01 | 0.0000 | 3027.0 | 24.73% | 8764.6 | 22.72% | motif file (matrix) | svg |
| 63 | C T G A C A T G A C T G A C G T A T G C C G T A C A T G T A C G A T G C G C T A T A C G C T G A C T A G A C T G A C G T A T G C C G T A T A G C | RAR:RXR(NR),DR5/ES-RAR-ChIP-Seq(GSE56893)/Homer | 1e-6 | -1.551e+01 | 0.0000 | 87.0 | 0.71% | 151.0 | 0.39% | motif file (matrix) | svg |
| 64 | C A T G A C T G A G C T A T G C C G T A A T G C G T A C G A C T T A C G C T G A A C T G A C T G G C A T A T G C C T G A | THRb(NR)/HepG2-THRb.Flag-ChIP-Seq(Encode)/Homer | 1e-6 | -1.491e+01 | 0.0000 | 1422.0 | 11.62% | 3944.3 | 10.23% | motif file (matrix) | svg |
| 65 | A G C T A G C T C A T G C T G A G T A C A G T C A G C T A G C T C A G T C T A G | RARa(NR)/K562-RARa-ChIP-Seq(Encode)/Homer | 1e-6 | -1.455e+01 | 0.0000 | 6771.0 | 55.31% | 20480.4 | 53.10% | motif file (matrix) | svg |
| 66 | T G C A C G T A G T C A A G C T A G T C G C T A T A G C C G A T C T A G G A T C | Gfi1b(Zf)/HPC7-Gfi1b-ChIP-Seq(GSE22178)/Homer | 1e-6 | -1.423e+01 | 0.0000 | 2553.0 | 20.85% | 7372.8 | 19.12% | motif file (matrix) | svg |
| 67 | G C A T C G T A C G A T A C T G A G T C G C T A C T G A C G T A C A G T A C T G C G T A T C A G | Oct6(POU,Homeobox)/NPC-Pou3f1-ChIP-Seq(GSE35496)/Homer | 1e-6 | -1.402e+01 | 0.0000 | 2060.0 | 16.83% | 5880.1 | 15.25% | motif file (matrix) | svg |
| 68 | C T A G C A T G A C G T A G T C G C T A A G C T A G T C A G C T T C A G C T G A A C T G C A T G G C A T A T G C C G T A | THRa(NR)/C17.2-THRa-ChIP-Seq(GSE38347)/Homer | 1e-5 | -1.285e+01 | 0.0000 | 1115.0 | 9.11% | 3074.0 | 7.97% | motif file (matrix) | svg |
| 69 | A T G C A T C G T A C G A G C T A T C G C T G A A G T C C T A G A G C T A T G C C T G A A T G C | CRE(bZIP)/Promoter/Homer | 1e-5 | -1.251e+01 | 0.0000 | 703.0 | 5.74% | 1869.9 | 4.85% | motif file (matrix) | svg |
| 70 | G C T A A C T G T C G A C T G A C T G A A C G T T A G C C T G A C G T A C G A T | Cux2(Homeobox)/Liver-Cux2-ChIP-Seq(GSE35985)/Homer | 1e-5 | -1.205e+01 | 0.0000 | 1465.0 | 11.97% | 4134.7 | 10.72% | motif file (matrix) | svg |
| 71 | G A C T C G T A A C G T A C T G A G T C C G T A C T G A C G T A C A G T A C G T G T C A T C A G | Brn1(POU,Homeobox)/NPC-Brn1-ChIP-Seq(GSE35496)/Homer | 1e-5 | -1.167e+01 | 0.0001 | 1596.0 | 13.04% | 4537.1 | 11.76% | motif file (matrix) | svg |
| 72 | C T A G C A G T C G T A A C G T A G T C A C T G C G T A A G C T A G T C G A T C | HNF6(Homeobox)/Liver-Hnf6-ChIP-Seq(ERP000394)/Homer | 1e-4 | -1.150e+01 | 0.0001 | 1739.0 | 14.21% | 4973.5 | 12.89% | motif file (matrix) | svg |
| 73 | C A T G G T A C C G T A A G T C C T A G A C G T A C T G G T A C A G T C A G C T | bHLHE40(bHLH)/HepG2-BHLHE40-ChIP-Seq(GSE31477)/Homer | 1e-4 | -1.121e+01 | 0.0001 | 954.0 | 7.79% | 2628.4 | 6.81% | motif file (matrix) | svg |
| 74 | C G T A C G T A C G T A G C A T G C A T A C T G G T A C G A C T C T A G G C T A T A C G G A C T T G A C C G T A A G C T | NFE2L2(bZIP)/HepG2-NFE2L2-ChIP-Seq(Encode)/Homer | 1e-4 | -1.099e+01 | 0.0001 | 348.0 | 2.84% | 872.9 | 2.26% | motif file (matrix) | svg |
| 75 | T A C G T C A G A G C T A T G C C G T A A G T C T C A G A C G T A C T G T C G A | USF1(bHLH)/GM12878-Usf1-ChIP-Seq(GSE32465)/Homer | 1e-4 | -1.070e+01 | 0.0001 | 1435.0 | 11.72% | 4076.9 | 10.57% | motif file (matrix) | svg |
| 76 | A T G C A G T C C T G A A G T C C G A T A C G T A G T C A G T C A C G T A T C G G A C T A C G T | Etv2(ETS)/ES-ER71-ChIP-Seq(GSE59402)/Homer | 1e-4 | -1.014e+01 | 0.0002 | 3336.0 | 27.25% | 9904.6 | 25.68% | motif file (matrix) | svg |
| 77 | G T C A A C G T C T A G G T C A C G T A G C A T C G T A C G T A G C A T C A G T A G T C C G T A C A G T C T A G C T G A | OCT:OCT(POU,Homeobox,IR1)/NPC-Brn2-ChIP-Seq(GSE35496)/Homer | 1e-4 | -9.778e+00 | 0.0003 | 124.0 | 1.01% | 270.1 | 0.70% | motif file (matrix) | svg |
| 78 | A G T C C T G A A G T C C G A T C A G T G A T C A T G C A C T G A T C G G A C T | Fli1(ETS)/CD8-FLI-ChIP-Seq(GSE20898)/Homer | 1e-4 | -9.585e+00 | 0.0004 | 3662.0 | 29.91% | 10934.3 | 28.35% | motif file (matrix) | svg |
| 79 | T C G A C A T G C A T G A C G T A T G C T C G A C T G A A G C T T A C G T G C A G T A C G A T C A G C T A G T C | FXR(NR),IR1/Liver-FXR-ChIP-Seq(Chong\_et\_al.)/Homer | 1e-4 | -9.468e+00 | 0.0004 | 943.0 | 7.70% | 2631.3 | 6.82% | motif file (matrix) | svg |
| 80 | C T A G A T G C A T G C C G A T A C T G G A C T A T G C G C T A T G A C A G C T T A G C G C T A | PBX1(Homeobox)/MCF7-PBX1-ChIP-Seq(GSE28007)/Homer | 1e-3 | -8.777e+00 | 0.0008 | 371.0 | 3.03% | 965.0 | 2.50% | motif file (matrix) | svg |
| 81 | T G C A C T G A A G T C G T C A A C T G A C T G C G T A C G T A C T G A A G C T | EWS:FLI1-fusion(ETS)/SK\_N\_MC-EWS:FLI1-ChIP-Seq(SRA014231)/Homer | 1e-3 | -8.132e+00 | 0.0016 | 2121.0 | 17.33% | 6236.3 | 16.17% | motif file (matrix) | svg |
| 82 | G A T C T C G A A G T C C G A T C G A T A G T C A T G C A C T G A T C G G A C T | Elk1(ETS)/Hela-Elk1-ChIP-Seq(GSE31477)/Homer | 1e-3 | -8.019e+00 | 0.0018 | 1584.0 | 12.94% | 4600.7 | 11.93% | motif file (matrix) | svg |
| 83 | G C A T T C G A C T G A G A T C A G T C G A T C G T C A G T C A A C G T A G T C C G T A C T G A | Duxbl(Homeobox)/NIH3T3-Duxbl.HA-ChIP-Seq(GSE119782)/Homer | 1e-3 | -7.977e+00 | 0.0018 | 272.0 | 2.22% | 693.4 | 1.80% | motif file (matrix) | svg |
| 84 | G T C A G C A T G C T A C A G T C T A G G A T C C G T A C T G A C G T A C G A T | Oct2(POU,Homeobox)/Bcell-Oct2-ChIP-Seq(GSE21512)/Homer | 1e-3 | -7.797e+00 | 0.0022 | 1378.0 | 11.26% | 3981.9 | 10.32% | motif file (matrix) | svg |
| 85 | T A C G A T G C G A C T A C T G A G C T A G T C G T C A T G C A A C G T A G T C G C T A T G C A | Pknox1(Homeobox)/ES-Prep1-ChIP-Seq(GSE63282)/Homer | 1e-3 | -7.510e+00 | 0.0028 | 899.0 | 7.34% | 2544.1 | 6.60% | motif file (matrix) | svg |
| 86 | T C G A G C A T A C G T C T A G G T A C T C G A G C A T T G A C T C G A A C G T | Chop(bZIP)/MEF-Chop-ChIP-Seq(GSE35681)/Homer | 1e-3 | -7.388e+00 | 0.0032 | 884.0 | 7.22% | 2502.5 | 6.49% | motif file (matrix) | svg |
| 87 | A C G T T G C A A G C T G A T C C T A G C T G A A G C T G T C A T C G A C G T A | CUX1(Homeobox)/K562-CUX1-ChIP-Seq(GSE92882)/Homer | 1e-3 | -7.219e+00 | 0.0037 | 2183.0 | 17.83% | 6459.3 | 16.75% | motif file (matrix) | svg |
| 88 | G C A T C G T A C G A T G A C T A C T G C T G A G A C T G A T C | Hnf6b(Homeobox)/LNCaP-Hnf6b-ChIP-Seq(GSE106305)/Homer | 1e-2 | -6.882e+00 | 0.0051 | 2749.0 | 22.46% | 8217.1 | 21.30% | motif file (matrix) | svg |
| 89 | A C T G C G T A A C T G A T G C T G A C G A T C A T C G T G C A A C T G A G T C | ZNF519(Zf)/HEK293-ZNF519.GFP-ChIP-Seq(GSE58341)/Homer | 1e-2 | -6.759e+00 | 0.0057 | 568.0 | 4.64% | 1574.5 | 4.08% | motif file (matrix) | svg |
| 90 | T G C A A T G C A C G T A C G T A C G T A T G C C T A G A C G T A C G T A G C T G A T C A G C T | T1ISRE(IRF)/ThioMac-Ifnb-Expression/Homer | 1e-2 | -6.529e+00 | 0.0071 | 55.0 | 0.45% | 112.3 | 0.29% | motif file (matrix) | svg |
| 91 | C G A T C T A G C G T A G A C T C A G T C T A G C G T A A G C T C A T G C T A G | HOXA1(Homeobox)/mES-Hoxa1-ChIP-Seq(SRP084292)/Homer | 1e-2 | -6.526e+00 | 0.0071 | 985.0 | 8.05% | 2828.8 | 7.33% | motif file (matrix) | svg |
| 92 | T A G C T A G C G A C T C T A G A G C T A G T C G T C A T G C A A C G T A T G C G C T A T G C A | Pbx3(Homeobox)/GM12878-PBX3-ChIP-Seq(GSE32465)/Homer | 1e-2 | -6.442e+00 | 0.0076 | 968.0 | 7.91% | 2779.8 | 7.21% | motif file (matrix) | svg |
| 93 | T C G A T A G C T G C A A C T G A C T G C G T A C G T A C T A G G A C T T A C G | ETS1(ETS)/Jurkat-ETS1-ChIP-Seq(GSE17954)/Homer | 1e-2 | -5.986e+00 | 0.0119 | 3575.0 | 29.20% | 10821.5 | 28.06% | motif file (matrix) | svg |
| 94 | C T G A C T G A C T A G T C G A C G T A A T G C C G T A A C T G C G T A A C G T C T G A C G A T A G C T C G T A A C G T A G T C C G A T T A C G G T C A G C A T | GATA(Zf),IR3/iTreg-Gata3-ChIP-Seq(GSE20898)/Homer | 1e-2 | -5.957e+00 | 0.0121 | 619.0 | 5.06% | 1743.9 | 4.52% | motif file (matrix) | svg |
| 95 | C T A G T A C G G A T C G T C A T G C A A C G T T G C A G C T A T C G A T G C A | Hoxa9(Homeobox)/ChickenMSG-Hoxa9.Flag-ChIP-Seq(GSE86088)/Homer | 1e-2 | -5.785e+00 | 0.0142 | 7824.0 | 63.91% | 24188.4 | 62.71% | motif file (matrix) | svg |
| 96 | G C T A A C G T C T A G G T C A C G T A A C G T C G T A C G A T C A G T A G T C C G T A A C G T C T A G C T G A A T C G | OCT:OCT(POU,Homeobox)/NPC-Brn1-ChIP-Seq(GSE35496)/Homer | 1e-2 | -5.762e+00 | 0.0144 | 74.0 | 0.60% | 166.6 | 0.43% | motif file (matrix) | svg |
| 97 | T C A G A G C T A T G C C G T A A G T C T C A G A C G T A T C G T C G A A G T C G A T C T G A C | TFE3(bHLH)/MEF-TFE3-ChIP-Seq(GSE75757)/Homer | 1e-2 | -5.651e+00 | 0.0159 | 290.0 | 2.37% | 777.2 | 2.02% | motif file (matrix) | svg |
| 98 | C G A T C T A G T C G A A G C T C G A T C T G A C G T A A G C T A C T G C T A G A T G C G A T C | Hoxb4(Homeobox)/ES-Hoxb4-ChIP-Seq(GSE34014)/Homer | 1e-2 | -5.451e+00 | 0.0193 | 782.0 | 6.39% | 2246.0 | 5.82% | motif file (matrix) | svg |
| 99 | A T C G A G C T C T G A C T A G A C T G A C G T G T A C G C T A A T G C A C G T C T A G C A T G T A C G C G A T A T G C C G T A | Reverb(NR),DR2/RAW-Reverba.biotin-ChIP-Seq(GSE45914)/Homer | 1e-2 | -5.396e+00 | 0.0202 | 364.0 | 2.97% | 998.8 | 2.59% | motif file (matrix) | svg |
| 100 | C T A G T A C G G A T C G T A C C T G A A G C T T G C A G C T A C G T A G C A T G A T C G C T A | Hoxc9(Homeobox)/Ainv15-Hoxc9-ChIP-Seq(GSE21812)/Homer | 1e-2 | -5.365e+00 | 0.0206 | 2013.0 | 16.44% | 6010.7 | 15.58% | motif file (matrix) | svg |
| 101 | C T G A T A G C T G A C T C A G C T A G G T C A C G T A T C A G A G C T T C A G | ETV4(ETS)/HepG2-ETV4-ChIP-Seq(ENCODE)/Homer | 1e-2 | -5.252e+00 | 0.0228 | 3424.0 | 27.97% | 10389.4 | 26.94% | motif file (matrix) | svg |
| 102 | G A T C C T G A A G T C C G A T C G A T G A T C A G T C A C T G A T C G A G C T | Elk4(ETS)/Hela-Elk4-ChIP-Seq(GSE31477)/Homer | 1e-2 | -4.926e+00 | 0.0313 | 1596.0 | 13.04% | 4745.3 | 12.30% | motif file (matrix) | svg |
| 103 | C G T A A C G T A G C T C G A T C T A G G T A C C G T A A G C T C G T A G C T A | Oct4(POU,Homeobox)/mES-Oct4-ChIP-Seq(GSE11431)/Homer | 1e-2 | -4.746e+00 | 0.0371 | 2139.0 | 17.47% | 6427.4 | 16.66% | motif file (matrix) | svg |
